# National health surveys: overview of sampling techniques and data collected using complex designs

**DOI:** 10.1590/S2237-96222023000300014.EN

**Published:** 2023-11-27

**Authors:** Célia Landmann Szwarcwald

**Affiliations:** 1 Fundação Oswaldo Cruz, Instituto de Comunicação e Informação Científica e Tecnológica em Saúde, Rio de Janeiro, RJ, Brazil

**Keywords:** Health Surveys, Simple Random Sampling, Cluster Sampling, Social Networking, Encuestas de Salud, Muestreo Aleatorio Simple, Muestreo por Conglomerados, Red Social, Inquéritos de Saúde, Amostragem Aleatória Simples, Amostragem por Conglomerados, Rede Sociais

## Abstract

This article aimed to present an overview of national health surveys, sampling techniques, and components of statistical analysis of data collected using complex sampling designs. Briefly, surveys aimed at assessing the nutritional status of Brazilians and maternal and child health care were described. Surveys aimed at investigating access to and use of health services and funding, those aimed at surveillance of chronic noncommunicable diseases and associated behaviors, and those focused on risk practices regarding sexually transmitted infections were also addressed. Health surveys through social networks, including online networks, deserved specific attention in the study. The conclusion is that the development of health surveys in Brazil, in different areas and using different sampling methodologies, has contributed enormously to the advancement of knowledge and to the formulation of public policies aimed at the health and well-being of the Brazilian population.

## Resumo

O artigo teve por finalidade apresentar um panorama dos inquéritos nacionais de saúde, técnicas de amostragem e componentes da análise estatística de dados coletados por desenhos complexos de amostragem. Foram descritos, brevemente, os inquéritos dirigidos à avaliação do estado nutricional dos brasileiros e da atenção à saúde materno-infantil. Inquéritos voltados à investigação do acesso, utilização dos serviços e financiamento da saúde, aqueles dedicados à vigilância das doenças crônicas não transmissíveis e comportamentos associados e os focados nas práticas de risco às infecções sexualmente transmissíveis foram também abordados. As pesquisas de saúde por redes sociais, incluindo as virtuais, mereceram atenção específica. Conclui-se que o desenvolvimento de inquéritos de saúde no Brasil, em diferentes áreas e por distintas metodologias de amostragem, contribuiu enormemente para o avanço do conhecimento e a formulação de políticas públicas dirigidas à saúde e bem-estar da população brasileira.

## Resumen

Este estudio tuvo como objetivo presentar una descripción de las encuestas nacionales de salud, las técnicas de muestreo y los componentes del análisis estadístico de diseños de muestreo complejos. Brevemente, se describieron encuestas destinadas a evaluar el estado nutricional y la atención a la salud materno-infantil. También se abordaron las encuestas dirigidas a investigar el acceso y uso de los servicios de salud y el financiamiento, las dirigidas a la vigilancia de las enfermedades crónicas no transmisibles y comportamientos asociados, y las enfocadas a las prácticas de riesgo para las infecciones de transmisión sexual. Las encuestas de salud a través de las redes sociales, incluidas las virtuales, merecieron atención específica en el estudio. Se concluye que el desarrollo de encuestas de salud en Brasil ha contribuido enormemente para el avance del conocimiento y para la formulación de políticas públicas dirigidas a la salud y el bienestar de la población brasileña.

## INTRODUCTION

Health surveys are characterized by being cross-sectional studies that provide information about the health situation of a given population, through the collection of information from a sample of individuals at a specific point (period) in time. Generally, data is collected through interviews - and/or other instruments - with the individuals selected to comprise the sample, in order to capture health information. Statistical methods are then used with the purpose of expanding the results to the total population studied.^1^ ^),(^ ^2^

Depending on the main object of research, the data collected addresses different dimensions related to the health situation. In addition to information on health conditions and risk and protective factors related to the conditions considered in a given study, health surveys enable questions about care provided, such as access to and use of health services, health funding and health service user satisfaction.^3^ ^),(^ ^4^

Health surveys are of great importance, not only because they provide fundamental information for understanding the population’s health situation and for assessing current health policies, but also for monitoring progress made towards meeting national and global health goals.^5^ ^),(^ ^6^ Furthermore, investigating the various dimensions of health, together with the demographic characteristics, socioeconomic and environmental conditions of the individuals researched, complements knowledge about health inequalities and thus enables guidance and planning of actions to achieve greater equity in the services provided to the population.^7^ ^),(^ ^8^

The objective of this article was to present an overview of health surveys in Brazil, the different sampling techniques used, as well as methods for statistical analysis of data collected using complex sampling designs.

## NATIONAL HEALTH SURVEYS

The first household-based survey registered in Brazil involving a health-related topic was the National Family Expenditure Study (*Estudo Nacional da Despesa Familiar* - ENDEF), carried out by the Brazilian Institute of Geography and Statistics (*Instituto Brasileiro de Geografia e Estatística* - IBGE) in 1974-1975. In addition to food consumption and family expenditure on food, the ENDEF obtained anthropometric data that made it possible to assess the nutritional status of the population and establish the prevalence of child malnutrition according to Brazil’s macro-regions. The main objective of the survey known as the National Health and Nutrition Survey (*Pesquisa Nacional sobre Saúde e Nutrição* - PNSN), conducted in 1989, was to estimate indicators for evaluating the nutritional status of the Brazilian population, in order make a comparison with the results obtained through the ENDEF.^9^

With the aim of investigating the food consumption and expenses of the Brazilian population, since 1987-1988 the IBGE has conducted the Family Budget Survey (*Pesquisa de Orçamentos Familiares* - POF) every six to seven years, monitoring the households comprising the sample over a 12-month period. The first three editions of the POF (1987-1988; 1995-1996; 2002-2003), as well as the 2017-2018 POF survey, provided information on food available in households.^10^ The 2002-2003 and 2008-2009 surveys enabled investigation of overweight/obesity trends through anthropometric measurements of those living in the sample households.^11^

Demographic and health surveys (DHS) began in 1984. Today, implemented in 90 countries, these surveys constitute an important source of health information on maternal and child care.^12^ In Brazil, the DHS goes by the name of the National Demographic and Health Survey (*Pesquisa Nacional de Demografia e Saúde* - PNDS). Conducted in 1986, 1996 and 2006, the PNDS enabled assessment of changes in women’s and children’s health,^13^ as well as enabling international comparisons.^14^Still with regard to ​​maternal and child health, the Being Born in Brazil - National Survey on Child Delivery and Birth (*Nascer no Brasil - Inquérito Nacional sobre Parto e Nascimento*) was conducted in 2011-2012 with the aim of investigating antenatal care, child delivery, birth and the postpartum period.^15^ Recently, the National Child Food and Nutrition Study (*Estudo Nacional de Alimentação e Nutrição Infantil* - ENANI) concentrated on researching eating patterns, nutritional status and micronutrient deficiency in children under 5 years old.^16^

In 1998, through a joint initiative between the IBGE and the Ministry of Health, a complementary module, with questions about access to and use of health services, was added to the National Household Sample Survey (*Pesquisa Nacional de Amostra por Domicílios* - PNAD). Known as the PNAD Health Supplement, it was conducted in 2003 and 2008, enabling the monitoring of several health indicators;^17^ in 2008, for example, the Special Smoking Survey (*Pesquisa Especial de Tabagismo*- PETab) was conducted with a PNAD subsample and investigated important aspects related to tobacco smoking.^18^

In 2001, the World Health Organization (WHO) proposed the carrying out of the World Health Survey (WHS) with the purpose of evaluating the performance of health systems.^19^ This process encouraged the WHS being conducted in several countries, thus enabling global comparison of important health indicators.^20^ In Brazil, the WHS was conducted in 2003 and addressed questions about diagnosis and care for six chronic noncommunicable diseases (NCDs), healthy habits as protection against NCDs, medication use, having private health insurance and health service user satisfaction with care provided.^21^

Due to the continuous growth of NCDs in Brazil,^22^ the Ministry of Health has developed within its Health and Environment Surveillance Secretariat a system especially dedicated to NCD surveillance, based on the periodic carrying out of a variety of health surveys.^23^ Taken together, the information collected through these surveys makes up a population-based information system for surveillance of several NCDs and the main health behaviors associated with them.^24^

In 2003, a household survey was conducted to collect information on the main NCDs and associated factors. The research, coordinated by the National Cancer Institute (*Instituto Nacional do Câncer* - INCA), was carried out in 15 Brazilian state capitals and the Federal District.^25^

In 2006, the Chronic Disease Risk and Protective Factors Surveillance System Telephone Survey (*Sistema de Vigilância de Fatores de Risco e Proteção para Doenças Crônicas não Transmissíveis por Inquérito Telefônico* - VIGITEL) was implemented,^26^ with the aim of monitoring prevalence of NCD-associated health behaviors in all the Brazilian state capitals. The VIGITEL survey has been carried out annually (2006-2022), through telephone interviews with at least 2,000 individuals aged 18 or over in each state capital. The annual information produced by VIGITEL has proven to be fundamental for analyzing the adoption of healthy and unhealthy behaviors in Brazilian state capitals, in addition to contributing to health promotion actions.^27^

With regard to health behaviors among adolescents, the National School Student Health Survey (*Pesquisa Nacional de Saúde do Escolar* - PeNSE) was carried out in 2009, 2012, 2015 and 2019. It is a survey with students from public and private schools in the state capitals and the Federal District, conducted jointly by the Ministry of Health, the Ministry of Education and the IBGE.^28^ With four editions already having been carried out at the time of this publication, the PeNSE survey reaffirms itself as an important instrument for the sustainability of the surveillance system regarding the lifestyles of high school students, as well as providing input for health service managers in planning actions aimed at Brazilian adolescents to prevent diseases and illnesses and promote health.^29^ Complementary to this, the Adolescent Cardiovascular Risks Study (*Estudo dos Riscos Cardiovasculares em Adolescentes* - ERICA), a survey that began in 2008, based on a sample of adolescents aged 12 to 17 enrolled at public and private schools, was designed with the purpose of estimating prevalence of diabetes *mellitus* and obesity, as well as assessing risk of cardiovascular diseases in this age group.^30^

Through yet another partnership between the IBGE and the Ministry of Health, the National Health Survey (*Pesquisa Nacional de Saúde* - PNS), which is household-based and uses probabilistic sampling at all selection stages, was conducted for the first time in 2013. The PNS sample size was calculated with a view to estimating several indicators, according to Federative Units, state capitals and metropolitan regions. The PNS design was based on three fundamental axes: (i) evaluation of the national health system, (ii) the population’s health status and (iii) surveillance of NCDs and associated health behaviors, in addition to aspects related to equity in health conditions and health service distribution.^31^

The second edition of the PNS, carried out in 2019, retained most of the modules covered in the first edition but also included new ones, such as communicable diseases and sexual activity.^32^ The PNS conducted 64,348 and 94,114 home interviews in 2013 and 2019, respectively.^33^

A relevant aspect of the PNS has been its measuring weight and height, these being fundamental markers for monitoring overweight and obesity trends in the Brazilian population.^34^ The 2013 PNS also included the collection of biological material (blood and urine), under the responsibility of a consortium of private laboratories.^35^ Laboratory testing was performed on 8,952 individuals, after which the results were analyzed to determine the prevalence of several health conditions, including cholesterolemia, diabetes *mellitus*, chronic kidney disease and anemia, among other health conditions.^36^ ^),(^ ^37^

In the field of communicable diseases, several surveys have been conducted with effect from the 1990s to monitor behaviors associated with HIV infection and other sexually transmitted infections (STIs). Thanks to a joint initiative between the Ministry of Health and the Ministry of Defense, research has been carried since 1996 out among Brazilian Armed Forces conscripts, allowing the periodic assessment of sexual practices among young men aged 17 to 20 years old.^38^ In 2004, the first edition of the survey entitled Knowledge, Attitudes and Practices of the Brazilian Population (*Pesquisa de Conhecimentos, Atitudes e Práticas da População Brasileira* - PCAP) was carried out to investigate forms of HIV transmission, STI risk practices and periodic HIV and syphilis testing. Further editions of the same survey conducted in 2008 and 2013 were an opportunity for collecting and providing information for developing indicators for monitoring and evaluating strategies to prevent these infections among the Brazilian population.^39^

## STATISTICAL METHODS USED IN SURVEYS

According to statistical concepts, a “target population” is considered to be the group of people that is being studied; while a population “sample” is a subset of people selected from within a target population. A sample is said to be probabilistic when each individual in the population under study has a known probability - greater than zero and less than or equal to 1 - of being selected.^40^

Simple random sampling, where each individual in the population has an equal and independent probability of being selected, can be used in surveys that have a list of sampling units and do not require interviewers displacement, such as telephone surveys. In household surveys, given the operational and cost difficulties, a set of probabilistic methods is used to provide a representative sample of the population, within the time period and budget foreseen when planning the research.^1^

There are several probabilistic sampling techniques, the most commonly used being simple random sampling, stratified random sampling, systematic random sampling and cluster random sampling. Stratified sampling presupposes the division of the population into homogeneous subgroups, according to geographic and sociodemographic characteristics, called “strata”. A sample is selected separately in each stratum. In systematic random sampling, widely used for studies of electronic medical records, a random selection of the first element is carried out and then subsequent items are selected using a pre-ordered periodic system.^1^ ^),(^ ^2^

In cluster sampling, the sampling unit is made up of a set of individuals from the population. To make fieldwork feasible, this type of sampling is repeated in several stages. Clusters are units composed of subunits, to be selected at each stage until reaching the survey respondents. At each stage, any of the probability sampling methods can be used to select sampling subunits.^1^ ^),(^ ^2^ For example, in the first stage of the PNS, census tracts (or a set of tracts) are selected, called “primary sampling units” (PSUs). In the second stage, households are selected in each PSU, and in the third stage, the resident of the household who will answer the individual questionnaire is selected.^33^

The use of various probabilistic sampling methods to select a representative sample of the population, referred to as “complex sampling design”,^41^ requires that statistical data analysis must take into account the complex sampling elements.^42^ ^),(^ ^43^

With the aim of expanding sample estimates to the general population, so-called “natural expansion factors” (weighting) are used, calculated by taking the inverse of the product of the selection probabilities at each stage. In order to correct the natural factors of the design, when the respondent is absent or refuses to answer, the data needs to be calibrated using known totals of the population.^44^

In addition to weighting the database, cluster effects must be considered in the statistical analysis of data, since the dependence of observations within clusters affects variability measurements.^45^ In order to measure the sample design effect (Deff), the ratio between variance estimated by the sample design and variance estimated by a simple random sample of the same size is calculated. The Deff is used to check for loss of precision of the estimate, as well as to support sample size calculation in subsequent research.^33^

However, limitations in interpreting survey results do not solely relate to statistical analyses that fail to consider the sample design effect. The gathering of information on health problems and corresponding health behaviors in a cross-sectional study occurs simultaneously, as temporal bias can potentially undermine the analysis of associations between variables.^46^

## SOCIAL NETWORK SURVEYS

Among the characteristics of populations that are hard to reach, also called “hidden” populations, are the small number of people (rarity) and being spread over a large geographic area (dispersion).

Due to the difficulties of using traditional sampling methods in hard-to-reach populations, chain-referral sampling techniques have been developed. Chain-referral sampling is when members of the population group that is being studied invite peers from the same group to participate in the research. Chain-referral sampling is based on the following assumption: people with a certain characteristic or activity have links with other people with similar attributes, with whom they relate through a social network.^47^

The first chain-referral sampling method was the so-called “snowball” method, in which “seeds”, i.e. participants of the target population, are initially selected (by convenience) and invited to begin the recruitment process. These “seeds” invite peers from the same population group to make up the first wave of the recruitment network. In turn, those invited by the “seeds” recruit other peers, and so on. Participants generally recruit peers with characteristics similar to theirs, which can lead to overrepresentation of certain characteristics of recruited individuals, to the detriment of others.^47^

In order to compensate for uneven distribution of some sociodemographic characteristics, post-stratification procedures have been frequently used. In order to obtain a representative sample of the population, according to a set of variables, such as sociodemographic and geographic characteristics, weighting is performed, based on a population with known proportions for the same variables.^42^ A limitation of this method, however, lies in the selection of variables and the definition of analysis categories. The absence of variables associated with the outcome in the post-stratification procedure may affect the estimates of the indicators that are being studied.^48^

The Respondent-Driven Sampling (RDS) method, as proposed by Douglas Heckathorn in 1997,^49^ was designed to enhance the “snowball” sampling procedure. The RDS method begins with the non-random selection of individuals from the target population to participate in the study (“seeds”), precisely because they have an extensive social network of contacts. The “seeds” recruit a fixed number of acquaintances from the same population group, forming the first wave of the network of participants. These recruit other peers and so on, until a number of waves is created that is large enough to achieve representativeness between the different characteristics of the population and reach equilibrium, when the prevalence of the outcome remains constant in several consecutive waves.^50^

Since its creation, several procedures have been added to RDS, which has allowed it to be considered a method with a complex sampling design. Taking the hypothesis that the larger the size of an individual’s social network, the greater their likelihood of participating in the research, the weighting used is inversely proportional to the size of each participant’s network.^50^ In order to reduce peer recruitment bias, the number of invitations is fixed and limited: just two to five invitations per individual. Recruiter-recruitee links are identified by numerical codes, assigned to participants and those whom they invite, with the purpose of taking into account the dependence of observations in estimating the variance of indicators of interest.^51^

With regard to statistical analysis of data, the RDS data collection design is considered to be a cluster sample, composed of those recruited by each recruiter.^52^ This procedure is equivalent to RDS-II as per the RDS-Analyst software package, appropriate for populations of unknown size. In order to estimate the variance of the variables that are being studied, the bootstrap procedure is used: a large number of successive sample simulations, generated by a process analogous to the original one.^53^

In Brazil, a comprehensive understanding of this sampling technique has been achieved through the application of the RDS methodology in populations at higher risk for HIV.^54^

## ONLINE SOCIAL NETWORK SURVEYS

Surveys undertaken through online social networks are very promising for health research. Surveys using traditional sampling methods is not always feasible when there are limitations in time and resources for data collection, or when there is a lack of a referral list or system.^55^ Furthermore, restricted physical contact during the COVID-19 pandemic, combined with the need to obtain information about the disease quickly, encouraged research to be carried out via internet.^56^

In Brazil, with the aim of researching changes in the adoption of healthy behaviors and the health situation of the Brazilian population during the pandemic, the ConVid - Behavior Survey (*Pesquisa de Comportamentos*) was carried out using the “online snowball” method.^57^ In a similar way to “snowball” sampling of peers recruited by survey participants, the “online snowball” method begins with the sending of an invitation to participate in the survey, sent by email or online social network, containing a link to access an electronic questionnaire. In this message, the recipient is asked to share the invitation with their contacts. In turn, the people who are contacted invite others from their online social networks, and so on, until a sample large enough to estimate the variables of interest is formed.^57^

As the survey was carried out using a non-probability sampling technique, sample weights were estimated using a post-stratification procedure. Geographical variables (Federative Unit and capital/rest of the Federative Unit) and sociodemographic variables (sex, age group, level of education and race/skin color) were used, based on population estimates from the PNAD 2019. For both sociodemographic and geographic variables, the necessary level of diversity was obtained in order to enable data weighting. 

Although research via online social networks has a series of advantages, there are several limitations with regard to statistical analysis of the data: (i) people who do not have access to the internet are excluded from the sample; and as the participants are volunteers, (ii) it is not possible to estimate the selection probabilities or the non-response rate. Furthermore, as recruitment is driven by respondents, links between recruiter-recruitee peers are not reported, thus affecting adequate variance estimation.^57^

In 2023, in order to evaluate changes in health behaviors in the post-pandemic period, a new edition of the ConVid survey will be carried out using RDS to collect information, thus enabling design effect to be taken into consideration in the data analysis.

## FINAL CONSIDERATIONS

The gradual development of health surveys in Brazil, in different areas of knowledge and using different sampling methodologies, has contributed intensely to increasing knowledge and formulating public policies aimed at the health and well-being of the Brazilian population. Over the years, Brazilian researchers have taken on the methodology for conducting surveys, both with regard to sampling techniques and the development of questionnaires, as well as statistical inference in data collected using complex designs. 

All of these initiatives contribute enormously to the production of information capable of supporting health actions, as well as monitoring the achievement of national and global health goals. Surveys focusing on NCDs provide information for monitoring indicators of the Global Action Plan for the Prevention and Control of Noncommunicable Diseases. In the case of surveys aimed at communicable diseases, they make it possible to monitor progress in controlling the HIV epidemic, combating tuberculosis and eliminating neglected tropical diseases. This set of information is relevant for Brazil to evaluate public policies and achieve the Sustainable Development Goals (SDGs), in order to comply with the United Nations 2030 Agenda.

We conclude that this article, without having the intention of citing all health research carried out in Brazil or exhausting the topic of possible sampling methods, has fulfilled its objective of providing a brief presentation of national health surveys and the different techniques used for data collection, enabling the production of knowledge capable of supporting public health and welfare policies for the Brazilian nation.
